# Ankle instability and gait disturbance after free fibula flap reconstruction in head and neck cancer reconstruction: A systematic review

**DOI:** 10.1016/j.jpra.2025.08.005

**Published:** 2025-08-07

**Authors:** Sebastian Holm, Jenny Löfgren, Fredrik Landström, Rodi Ali, Reza Tabrisi, Alexander Wyckman, Nikoo Bazsefidpay, Johann Zdolsek, Juan Enrique Berner

**Affiliations:** a Department of Plastic and Reconstructive Surgery, Örebro University Hospital, Örebro, Sweden; b Faculty of Medicine and Health, Örebro University, Örebro, Sweden; c Department of Molecular Medicine and Surgery, Karolinska Institute, Stockholm, Sweden; d Department of Hand Surgery, Plastic Surgery and Burns and Department of Biomedical and Clinical Sciences, Linköping University, Linköping, Sweden; e Department of Orthopaedics in Norrköping, and Department of Biomedical and Clinical Sciences, Linköping University, Linköping, Sweden; f Division of Plastic Surgery, Department of Surgery, University of Texas Health Science Center, San Antonio, TX, USA; g Department of Otorhinolaryngology, Örebro University Hospital, Örebro, Sweden; h Department of Reconstructive Plastic Surgery, Karolinska University Hospital, Stockholm, Sweden; i Department of Oral and Maxillofacial Surgery, Örebro University Hospital, Örebro, Sweden

**Keywords:** Head and neck reconstruction, Microsurgery, Donor site morbidity, Ankle instability, Gait disturbance, Complications

## Abstract

**Introduction:**

The free fibula flap is a workhorse flap for bony reconstruction of the craniofacial skeleton. The aim of the study was to conduct a systematic review to investigate the postoperative donor site complications and functional outcomes, specifically ankle instability (AI) and gait disturbances (GD), for patients who have received a free fibula flap (FFF) for head and neck cancer reconstruction.

**Methods:**

We designed a PRISMA-compliant systematic review, which was registered prospectively in PROSPERO. Searches were designed with a health science librarian and included MEDLINE, EMBASE, CINAHL and PEDro. Risk of bias assessment was conducted for each included study, with an assessment of quality using GRADE.

**Results:**

Following exclusion of duplicate entries, a total of 1940 abstracts were identified. After parallel blinded eligibility assessment, 32 studies were included in the analysis. The total number of included participants was 1163, with the total number of FFF being 955. The mean time for functional assessment was 35 months postoperatively (range 8–81 months). The subjective and objective assessment modalities varied considerably. The primary result for AI were 3.3 % and 5.5 % for GD. The results demonstrate heterogeneity in the literature regarding the reporting of AI and GD following FFF.

**Conclusion:**

According to this review, the risk of developing these complications appears to be limited but underreporting may be a limitation. Consensus on methods for standardized outcomes assessment of FFF-reconstruction is needed to establish the impact of free fibula flap on AS and GD.

## Introduction

The free fibula flap (FFF) is a widely utilized option for restoring bony defects in head and neck cancer patients.^1^, ^2^, ^3^, ^4^ While high flap survival rates of 91.9–93 % have been reported,^5^^,^^6^ the harvesting procedure has an associated donor site morbidity. Reported long-term functional complications vary considerably, with documented issues including ankle instability, reduced range of motion (ROM), sensory deficits, hallux contracture, lesser toe deformities, gait disturbances, and leg weakness.^7^, ^8^, ^9^ Furthermore, valgus deformity of the ankle following flap harvest has been observed in paediatric patients.^10^ However, the extent and impact of these donor site morbidities is not clear.^11^^,^^12^

There are a variety of both objective and subjective methods for assessment of functional donor site morbidity. Examples of objective measurements include balance tests, gait analysis, electromyography, ROM and isokinetic strength tests while subjective measurements include validated patient-reported outcome measures (PROMs), ad-hoc questionnaires and aesthetic evaluation by the patients and/or surgeons.^13^, ^14^, ^15^, ^16^, ^17^ To our knowledge, no gold standard for functional assessment of the flap donor site has been established.

Gait is usually classified as normal or abnormal where abnormalities are categorized as major or minor. Major gait abnormalities disrupt the overall gait pattern while a minor gait abnormality involves subtle changes in stance or swing phases.^18^ The gait cycle consists of a 60 % stance phase (heel strike to toe off) and with a 40 % swing phase (acceleration to deceleration). The swing phase primarily engage hip, knee flexors, and the anterior tibial muscle.^18^

The aims of this study were to conduct a systematic review to investigate both the frequency of and the methods used for assessment including both postoperative ankle instability (AI) and gait disturbance (GD) in patients where a FFF was used for head and neck cancer reconstruction.

## Methods

### Search strategy

Search strategies were designed with the assistance of a health science librarian according to a pre-registered Prospero protocol CRD42024478655 and in adherence to PRISMA guidelines.^19^ The search strategy is found in Supplementary Table 1. The search was performed on February 5th, 2024, including the databases MEDLINE, EMBASE, CINAHL and PEDro. There were no language restrictions, no time limit and all types of study designs were included in the search.

### Eligibility criteria

Studies including patients undergoing head and neck reconstruction with a FFF where postoperative assessments of AI and GD were performed were included. Studies with other reconstructions not involving FFF, without gait assessment, patients undergoing pedicled fibula reconstruction, basic science/animal studies and literature reviews were excluded.

### Study selection

The screening was performed using Covidence (Covidence systematic review software, Veritas Health Innovation, Melbourne, Australia) for a parallel blinded peer review process. After the database search and removal of duplicate entries, the titles and abstracts were screened blindly for eligibility by two independent investigators (S.H. and R.T.). A first assessment was based on title and abstracts, followed by a second full text assessment for potentially eligible articles. Initial disagreements between the reviewers were resolved through discussion. However, a third reviewer (J.B.) was consulted when an agreement could not be reached.

### Data extraction

The data extraction was performed using a pre-defined Excel v 2408 (Microsoft Corp., Redmond, Washington, USA) spreadsheet. The information collected was first author, study country, type of study, number of patients included, mean age, unilateral/bilateral FFF, time of assessment, subjective assessment, objective assessment, results (subjective/objective), frequency of AI and GD (subjective/objective).

### Quality assessment

Methodological quality assessment for the final list of eligible studies was conducted by two separate investigators in an independent manner. The applicability and reliability of the research were assessed by the Joanna Briggs Institute (JBI) critical appraisal tool checklist.^20^ The JBI total quality score varies between 0 and 100 %, where 71–100 % = low risk for bias; 50–70 % = moderate risk and 0–50 % = high risk. Disagreement between the two primary reviewers were resolved through discussion with a third investigator until agreement was reached.

The certainty of the evidence was assessed and performed using the Grading of Recommendations Assessment, Development, and Evaluation (GRADE) tool, classified into four levels: high, moderate, low, or very low.^21^

## Results

### Search and study selection

**Figure 1:**
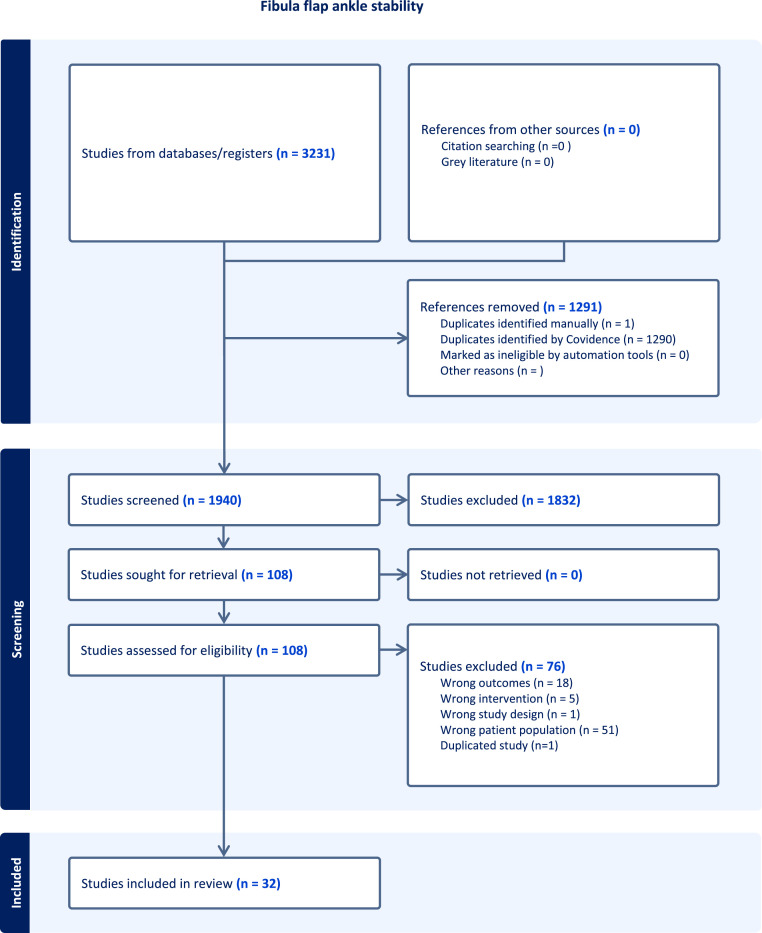
PRISMA flow diagram from Covidence.

After exclusion of duplicates 1940 abstracts remained for screening. Following eligibility assessment, 32 studies were included in the analysis. The Preferred Reporting Items for Systematic Reviews and Meta-Analyses (PRISMA) flow diagram can be found in Figure 1.

### Study and patient characteristics

Retrospective studies were most common (*n* = 14), followed by prospective cohort (*n* = 10) and cross-sectional studies (*n* = 3). The total number of included participants was 1163, with the total number of FFF being 955. The mean time for functional assessment follow-up was 35 months (8–81 months). The most common countries of origin of the published studies were Germany (*n* = 7), USA (*n* = 5) and Italy (*n* = 4) (Table 1).

**Table 1:** Data extraction table based on the 32 included articles in the systematic review.

Study	Country	Study design	Number of patients	Number of FFF	Age (mean in years)	Unilateral/bilateral FFF	Time of assessment (mean in months)	Subjective assessment	Objective assessment	Results (subjective/objective)	Ankle instability (nr of patients)	Gait disturbance (nr of patients)
1. Shindo et al.^15^	USA	RS^a^	53	53	54	Unknown	>3	Charts, questionnaires, interviews	Physical examination	Sensory/motor deficit, resulted in impaired gait/alteration in daily activities	0	3
2. Lin et al.^32^	Taiwan	CCS^b^	15 (8 controls)	14	53	Bilateral	34	Questionnaires (Point Evaluation System for Lower Extremity Fibulectomy)	• Balance test (dynamic/static), SMART Balance Master (NeuroCom International, Inc, Clackamas, OR) • Gait analysis (VICON 370 motion system (Oxford Metrics, Oxford, UK + 2 AMTI (Model OR6–5–1000, Advanced Biomechanical Technology, Newton, MA)	Subjective: mild pain after prolonged standing and gait (*n* = 1), difficult in squatting (*n* = 2) and paraesthesia donor-site (*n* = 1) Objective: no significant difference with compared controls (dynamic balance test, static postural stability test, gait analysis)	0	0
3. Rendenbach et al.^14^	Germany	PS^c^	19	19	58	Unknown	Preoperative: one week Postoperative: 12	AOFAS^d^ score	• Jumping mechanography on LeonardoMechanograph® Ground Force Reaction Plate (Leonardo GFRP, Novo-tec Medical GmbH, Pforzheim, Germany) • Balance testing: ground force reaction plate • Esslinger Fitness Index (EFI), primary endpoint • AOFAS	No significant difference between pre- and postoperative EFI. AOFAS score reduced by 8.8 p^5^. Dorsal extension and flexion of ankle, significantly reduced.	0	4
4. Zavala et al.^42^	Peru	RS	34	34	10	Unknown	>12	Medical records	Physical examination	Normal mobilisation achieved after 12 days. Two patients with gait disturbances, required physiotherapy over 18M	0	2
5. Zimmermann et al.^8^	Germany	RS	42	42	48	Unilateral (*n* = 41) Bilateral (*n* = 1)	35	Patient interviews	Physical examination	Subjective: 76.3 % unlimited walking distance. 23.7 % could walk up to 15000 m. One patient used only one leg, all other climbed stairs. Discomfort in 21.1 %. Instability (*n* = 2). 31.6 % with restrictions (mobility, dorsal extension of foot, toe mobility, dorsal extension and plantar flexion. Walking disturbance (*n* = 3) Objective: 39.5 % foot and/or toe mobility limitations. 44.7 % reduced strength in foot and/or toes.	Subjective: 3 Objective: 7 (18,4 %)	Subjective: 3 Objective: 5
6. Momoh et al.^40^	USA	PS	157	157	53	Unknown	25	Medical records	Physical examination	Objective: long-term donor site morbidity in 17 %. Decreased ankle mobility (12 %) and ankle instability (4 %). Subjective: limitations in ankle range of motion (2 %)	6	0
7. Baj et al.^34^	Italy	CCS	8	8	55	Unknown	28	–	• Ascend and descend 3 steps. • Analysed movement by SMART optoelectronic computerized system (BTS, Milan, Italy)	Objective: % of swing, stride time and support did not differ. No functional limitations were found during stair ascend/descend.	0	0
8. Schardt et al.^22^	Germany	RS	46 (DCIS^e^*n* = 27, FFF *n* = 19)	19	61	Unknown	14	-SF-36 questionnaire (pain) -AOFAS score -Short form-36 Health Survey (SF-36) questionnaire (QoL^f^)	• Physical examination (motor and sensory function).	Objective: two patients (10.5 %) not able to walk/stand on heels. Mechanical stiffness to ankle (*n* = 9, 47.3 %). Weakness of dorsiflexion (*n* = 11, 57.8 %) Subjective: 68,42 % showed no impairment to stair climbing. *N* = 4 patients reported gait disturbance (slight-moderate)	0	Subjective: 4
9. Anthony et al.^41^	USA	PS	29	30	59	Unilateral: (*n* = 28) Bilateral: (*n* = 1)	7	Patient questionnaires	• Physical examination • Bilateral leg isokinetic testing	Objective: average ankle inversion + eversion range of motion decreased by 14 %. Strength of donor leg, significantly lower of all 4 ankle motions. Subjective: occasional ankle stability (*n* = 3, 10 %)	Subjective: 3 (10 %)	0
10. Lee et al.^18^	South Korea	PS	20	20	46	Unknown	3	–	Gait analysis (25 m walkway). Kinematic analysis: VICON system (Oxford Metrics, Oxford, UK) Kinetic analysis: two force plates (Advanced Medical Technology Incorporation, MA, USA)	Objective: decrease in time-distance, peak plantarflexion and dynamic range. No significant difference results in preoperative and postoperative gait analysis (3 M).	0	0
11. Xu et al.^16^	China	PS	30	30	46	Unilateral	12	Patient and Observer Scar Assessment Scale (POSAS) Patient complaints	Ankle joint strength by Isokinetic dynamometer (Biodex System (Biodex, New York, NY)) Electromyographic examination of donor calf Gait analysis by Footscan 9 system (RS-scan International, Belgium)	Objective: total work of ankle plantar flexion + dorsiflexion decreased. Subjective: weakness of lower extremity (16.67 %), swollen ankle after prolonged walking/standing (56.67 %)	0	0 (gait disturbances decreased after 3 M)
12. Syczewska et al.^33^	Poland	PS	30 (ICF^h^*n* = 14)	16	Unknown	Unknown	6	–	Gait analysis by VICON 460 and later using VICON MX system	Gait variables that changed postoperative: tilt, range of motion of tilt, time to maximum knee flexion and minimum hip flexion. The values of the gait lab showed normal limits. Gait velocity decreased after surgery, improved over time.	0	0
13. Macdonald et al.^37^	Canada	PS	8	Unknown	58	Unknown	3	Patient questionnaires (SCG^i^)	Gait analysis by Walkabout Portable Gait Monitor (WPGM)	Objective: no significant difference in velocity or step length 3 M postoperatively. Subjective: worsening ability to walk (*n* = 4), increased gait restrictions (*n* = 4).	0	0
14. Vittayakittipong^28^	Thailand	CSS^j^	17	17	40	Unilateral	12	Patient questionnaire Medical records Point evaluation system (PES) Visual analogue scale (VAS)	–	VAS: donor-site morbidity for gait alteration is considered low. PES: walking ability minor limitation (*n* = 2, 13,3 %). Gait alteration, minor (*n* = 13, 86,7 %) A significant positive correlation between average VAS and PES score.	0	0 (minor gait alteration and walking ability *n* = 15)
15. Pacifici et al.^35^	Italy	CS^k^	8	Unknown	Unknown	Unknown	6	–	Gait analysis by SMART optoelectronic computerized system (BTS spa, Milan)	No functional limitations were found.	0	0
16. Shpitzer et al.^7^	Canada	CS	47	Unknown	56	Unknown	17	Patient questionnaire	Physical examination	Subjective: weakness of great toe (*n* = 5, 12 %), ankle stability (*n* = 2, 5 %) Objective: restriction in ROM^l^ of ankle joint (*n* = 5, 12 %)	Subjective: 3 (7 %)	0
17. Rendenbach et al.^23^	Germany	PS	27	27	58	Unknown	8	AOFAS	- Jumping mechanography on Leonardo Mechanograph Ground Force Reaction Plate (Leonardo GFRP, Novotec Medical GmbH, Pforzheim, Germany) - Balance test	Subjective: decrease in ROM ankle joint and dorsal extension. Objective: reduced maximum peak power per body mass and balance. Did not reveal ankle instability.	0	Subjective: 11 (40.7 %)
18. Sieg et al.^17^	Germany	PS	57	62	Unknown	Unilateral: 57 Bilateral: 2	27	Kitaoka ankle-hindfoot score	Kitaoka ankle-hindfoot score	Objective: 3 pt (5 %) with abnormal gait. 4 pt with ankle instability.	Objective: 4 (7 %)	Objective: 3 (5 %)
19. Li et al.^38^	China	RS	45	Unknown	47	Unknown	Unknown	Medical record Patient questionnaire	–	Subjective: late dysfunction in 20 patients (57.1 %).	0	0
20. Di Giuli et al.^36^	Italy	LS^m^	14	14	50	Unknown	6	Questionnaires: WOMAC^n^and PESLEF^o^	Gait analysis by 9-camera optoelectronic movement analysis system (SMART-E; BTS, Milan, Italy)	Subjective: 10 pt (71 %) with no alterations on the donor leg. Objective: no significant difference in gait parameters, only 6 % reduction in double support phase. No alterations in ROM of joints.	0	0
21. Sugiura et al.^43^	Japan	RS	73	Older group: 17 Younger group: 20	Older group: 83 Younger group: 72	Unknown	6	Medical records Questionnaires: modified University of Washington Quality of Life assessment (UW-QOL)	–	Subjective: no patient in both groups reported gait disturbance or any major limitation of daily activities.	0	0
22. Santamaría et al.^44^	Mexico	RS	22	22	9,3	Unilateral: 21 Bilateral: 1	8,5	Medical records Questionnaire by Enneking and Bonde et al., modified by Sagalongos et al. (Sagalongos et al. 2011)	–	Subjective: gait alteration in 23 %, walking ability alteration 50 %,	0	Subjective: 5 (23 %)
23. Slijepcevic et al.^45^	USA	RS	87	87	12	unknown	unknown	Medical records	–	Subjective: gait abnormality was reported in one patient	0	1
24. Shah et al.^24^	UK	RS	26	26	49,4	unknown	14	Medical records AOFAS FADI^p^	SEBT^q^ by Olmsted et al. (Olmsted et al., 2002)	Subjective: claw toe (*n* = 5), numbness of superficial peroneal/sural nerves (*n* = 3) Objective: no sign of ankle instability.	0	0
25. Catalá-Lehnen et al.^25^	Germany	RS	42	42	55,5	unknown	81	Medical records AOFAS Short Form 36 Health Survey (SF-36)	–	Subjective: functional impairment (*n* = 11), reduced sagittal motion (*n* = 10), motor weakness (*n* = 2) and balance disturbance (*n* = 1).	0	Unknown
26.Ferrari et al.^29^	Italy	RS	5	10	46	Bilateral: 10	18,2	PES^r^	BESTest^s^	Subjective: most patients scored a 3–4, indicating gait was not affected. Objective: gait stability and sensory balance, minimally compromised.	0	0
27. Crosby et al.^39^	USA	RS	11 (children)	12	3,4	Unknown	9	Medical records	Physical examination	Objective: functional outcome —normal. Long-term malocclusion (*n* = 2). Great toe flexion contracture (*n* = 4). Valgus deformity (*n* = 1).	0	0
28. Attia et al.^31^	Germany	RS	68	68	55,4	Unknown	48	FADI	SEBT	Subjective: 72.1 % had some limitation from the donor site. FADI similar to chronic ankle instability results. Objective: average difference of 4,5 % between healthy and operated leg,	0	0
29. Maben, Anehosur, and Kumar^30^	India	PS	20	20	44,38	Unknown	unknown	PES	–	Subjective: major gait alteration at 15 days (75 %). Improvement after 6 M, 50 % with no gait deficit.	0	Subjective: 10
30. Ling, Peng, and Samma^26^	Malaysia	CS/RS	44	20	53,1	unknown	unknown	Patient interviews AOFAS The Harris Hip Score	Physical examination Stony Brook Scar Evaluation Scale	Subjective: no significant difference in functional loss, wound healing, pain, cosmetics. Objective: significant difference in dorsiflexion and plantar flexion.	0	Objective: 2
31. Farhadi et al.^27^	Switzerland	RS	48	48	55	Unilateral: 48	32,3	PES AOFAS	Physical examination Digital dynamic pedobarography system (Emed-AT System, Novel, Munich, Germany) Neuromuscular Biomechanical Analysis Radiologic evaluation	Subjective: 10 % major functional loss (*n* = 1) Objective: Pedobarography, showed decrease of big toe pushup force. Reduction of perioneus longus muscle on isometric strength measurements. 70 % with mild medial ankle osteoarthritis.	Subjective: 2 Objective: 4	0
32. Chou et al.^13^	Taiwan	CS	11	11	52,1	Unknown	27,4	Questionnaire	SMART Balance Master1 (NeuroCom international,Inc., Clackamas, OR) Static postural stability test Dynamic balance test Gait analysis by VICON 370 motion system	Subjective: no limitation in daily activities. None reported instability of ankle joint. Objective: no statistical of gait analysis, simple static balance test. Maximal stability of ankle, statistically different.	0	0

a Retrospective.

b Case-control study.

c Prospective study.

d American Orthopaedic Foot and Ankle Society.

e Points.

f Deep-circumflex iliac artery flap.

g Quality of life.

h Iliac crest flap.

i self-administered comorbidity questionnaire.

j Cross-sectional study.

k Case series.

l Range of motion.

m Longitudinal study.

n Western Ontario and McMaster Universities Osteoarthritis Index.

o Point Evaluation System for Lower Extremity Fibulectomy.

p Foot and Ankle Disability Index.

q Star excursion balance test.

r Point evaluation system.

s balance evaluation systems test.

### Subjective assessment

The subjective assessment methods for AI and GD varied between the included studies. The most common source were subjective entries from medical records (*n* = 10) and non-specific patient questionnaires (*n* = 8). Other studies used the following patient reported outcome measures (PROMs): The American Orthopaedic Foot and Ankle Society (AOFAS)^14^^,^^22^, ^23^, ^24^, ^25^, ^26^, ^27^ (*n* = 7), the Point evaluation system (PES)^27^, ^28^, ^29^, ^30^ (*n* = 4), SF-36 questionnaire^22^^,^^25^ (*n* = 2) and the Foot and Ankle Disability Index (FADI)^24^^,^^31^ (*n* = 2).

### Objective assessment

The reported objective methods for assessment of AI and GD with physical examination being the most common (*n* = 8), followed by gait analysis (*n* = 7) and star excursion balance test (*n* = 2). The tools for assessment of gait analysis varied where the VICON system (VICON Motion Systems Ltd, Oxford, UK)^18^^,^^32^^,^^33^ was used in some studies (*n* = 3) and the SMART optoelectronic computerized system (BTS Bioengineering, Milan, Italy) in others^34^, ^35^, ^36^ (*n* = 3) (p201). In one study a walkabout portable gait monitor^37^ was used.

### Outcomes

In total (subjective and objective) AI was found in 32 patients and GD in 53 patients. However, the subjective and objective assessment modalities varied considerably between the studies. Subjective AI was found in 11 patients (34.3 %) and objective AI in 15 patients (46.8 %). In 6 patients it was not clear whether it was based on subjective or objective assessment. Subjective or objective GD was documented in 10 out of 53 patients (18.8 %). Subjective GD was found in 33 patients (62.2 %). Objective GD was found in 10 patients (18.8 %). In 10 patients, it was not specified if subjective or objective GD (18.8 %) (see Table 2).

**Table 2:** Objective and subjective assessment of ankle instability and gait disturbance.

Assessments	Ankle instability	Gait disturbance
Subjective	11	33
Objective	15	10
Unknown	6	10
Total	32	53

In some patients the method of assessment was not clearly reported.

### Critical appraisal of studies

The included studies were critically assessed using the JBI critical appraisal tool checklist (Supplementary table 2–5) For the ten case series studies seven were evaluated as low risk of bias two as moderate risk and one as high risk.^7^^,^^17^^,^^23^^,^^26^^,^^27^^,^^29^^,^^35^^,^^36^^,^^38^^,^^39^ One case control study was evaluated as moderate risk.^13^ Of twelve cohort studies five^8^^,^^14^^,^^33^^,^^37^^,^^40^ were evaluated as low risk and seven^15^^,^^16^^,^^18^^,^^36^^,^^41^, ^42^, ^43^ as moderate risk. Seven of nine cross sectional studies^22^^,^^24^^,^^25^^,^^31^^,^^34^^,^^44^^,^^45^ were evaluated as low risk and two^28^^,^^30^ as moderate risk.

### Certainty of evidence

The evaluation of the quality of evidence was assessed using GRADE. Considering factors such as inconsistency, risk of bias, publication bias, imprecision, and indirect evidence. It was classified into four levels: high, moderate, low, or very low. (Supplementary Table 6) The majority of the studies (*n* = 21) showed a moderate risk of bias. For the evaluation of inconsistency it varied, some being moderate (*n* = 13), low (*n* = 8) and high (*n* = 6). Indirectness was largely moderate (*n* = 19). Imprecision was a common with majority as moderate (*n* = 12) and high (*n* = 6). For publication bias, a majority were evaluated as moderate (*n* = 15) and high (*n* = 13). For quality of evidence around 78 % (*n* = 25) were evaluated as moderate and 22 % (*n* = 7) of the studies evaluated as low.

## Discussion

This review reports an overall AI of 3.3 % and GD of 5.5 %, after FFF among 955 study participants who underwent a FFF reconstruction for head and neck cancer ablation. There was significant heterogeneity between the published studies where both the subjective and objective assessment methods varied as well as the time to follow up assessment (3–81 months).

The fibula does not substantially contributes to the weight bearing capacity of the lower extremity, however, it has a crucial role in providing stability to the ankle joint, by facilitating muscle attachments and aiding in load transfer.^46^^,^^47^ Current guidelines recommend keeping 6–8 cm of residual distal fibula after harvesting to ensure ankle stability.^7^^,^^41^^,^^46^^,^^48^^,^^49^ Pacelli et al.^48^ reported that ankle stability is possible with an even shorter residual fibula and their biomechanical testing suggested that AI was not evident until the residual length of the fibula fell below 10 % of its initial length. Their study suggests that ankle stability depends on the residual length of the fibula and stresses the importance of the stabilizing function of the syndesmotic ligaments for the ankle joint. Furthermore, it indicates that increased fibular movement (axial translation) may cause ankle pain.^48^ Contrastingly, Yang et al.^50^ suggests that preserving the proximal to middle third of the fibula is crucial for maintaining functional outcomes and resection of the middle or distal fibula can compromise the tibiotalar joint stability. It has also been suggested, that the proximal fibula also plays a crucial role in the kinetics of both the knee and ankle joints after resection of the fibula.^51^

Gait analysis results post-FFF reconstruction varied. Di Giuli et al.^36^ reported minimal gait changes, only 6 months after a FFF and that patients were generally unaware of the minor alteration, with high satisfaction despite their condition. Lee et al.^18^ instead indicated that the patients gait was normalized within three months post-FFF, with no long-term donor site abnormalities. Studies examining the gait one to three years after FFF, found minor gait differences as well.^9^^,^^13^ Two studies found no significant association between the fibula harvest length and gait asymmetry, suggesting the gait is not dependent on the resection-length of the fibula, compared to ankle instability.^52^^,^^53^ GD is not significantly impacted after a FFF, and full normalization of gait should be expected within a year according to existing literature.^13^^,^^53^^,^^54^Our review aligns with previous studies, with GD post-FFF reconstruction remaining low (5.5 %).

The reported subjective assessments used different questionnaires such as AOFAS, PES and SF-36. The objective assessment methods varied even more, where both physical examination and different technological systems (VICON, SMART or walkabout portable gait monitor) were used to evaluate gait. The timing for outcome assessment also varied where the most commonly reported times for donor site assessment were 6 and 12 months.

Subjective assessment with PROMSs can help define symptoms of a particular disorder and their impact on quality of life. Objective assessments typically involve physical measurement using standardised tests.^55^ A direct comparison was not possible in this study due to the wide variety of reported assessment methods and timings for outcome evaluation. It is impossible for the authors to comment how PROMs would theoretically correlate with objective assessment of donor site morbidity for patients who have undergone an FFF. The most common and validated PROM used, was the AOFAS score questionnaire. This is commonly used to establish the ankle joint function and is not primarily used to evaluate gait.^56^ The second most common was the Point evaluation system (PES), which is primarily used for to assess the functional status of the ankle joint, focusing more on pain, motion, stability and not intended to evaluate specific gait movements.^57^ This study demonstrates there is a more common use of subjective assessment when analysing AI. In contrast, to the objective assessment, the most common is physical examination (*n* = 8) for both AI and GD. The second most common objective assessment was the gait analysis (*n* = 8) with the majority using both a VICON system (*n* = 3) and a SMART optoelectronic computerized system (*n* = 3). The VICON system is widely known and considered as a gold standard for assessment and analysis of gait.^58^^,^^59^ The VICON system contain several infrared cameras which is tracking the movements with high accuracy.^60^^,^^61^ The SMART optoelectronic computerized system is also a system with infrared cameras and record at the same frequency.^62^ Both systems have a high accuracy in evaluating gait, thus VICON is considered superior.^61^

These results highlight the need for standardized donor site assessment protocols following FFF reconstruction.

For future assessment of donor site morbidity after a FFF, specifically regarding AI and GD there is a need for a combined and comparable evaluation. These should be assessed with a combination of both a subjective and an objective evaluation. The subjective assessment of AI is more important due to the difficulty to evaluate this condition clinically. For the evaluation of ankle instability, there is a need for a validated questionnaire such as AOFAS for future studies. AOFAS is not only a PROM alone with subjective findings, but also involves objective assessment from findings of a clinical examination. For GD, the subjective assessment is important, although we recommend primarily, an objective assessment of this condition. Due to the high accuracy of the VICON system,^61^ it is a good choice for establishing a coherent evaluation of gait disturbances after an FFF.

### Strengths and limitations

The critical appraisal of the studies revealed a moderate to low risk of bias across all included studies. The GRADE assessment demonstrated that the majority of included studies had moderate risk of bias, suggesting potential methodological weakness across the available evidence. Evaluation of inconsistency varied, potentially due to difference in interventions, populations and outcome measures. Indirectness was largely moderate, there was some aspects that made the findings less directly applicable such as intervention specifics or population characteristics. Imprecision was common due to wide confidence intervals, sample sizes and limited statistical power. It is our impression that the overall quality of the studies included in this review is restricted by various methodological limitations. To be able to draw definitive conclusion, future search should aim to improve consistency, precision and minimize biases.

There are several limitations in this systematic review. Acknowledging the limitations of the retrospective data, included information from case series and retrospective studies, some of which cover long periods and may reflect outdated surgical techniques, potentially introducing an additional cofounder. Due to the heterogeneity of the results, it was not possible to conduct a meta-analysis. The subjective and objective assessment varied which also affected the overall quality of evidence.

## Conclusion

The results demonstrate heterogeneity in studies reporting AI and GD in patients reconstructed with an FFF. According to this review, the reported number of patients suffering from these complications is low. This review highlights the need of a consensus regarding functional outcome assessment FFF reconstruction.

## Conflicts of interest

The authors declare that they have no known competing financial interests or personal relationships that could have appeared to influence the work reported in this paper.

## Appendix Supplementary materials

Image, application 1

Image, application 2

Image, application 3

Image, application 4

Image, application 5

Image, application 6

## References

[1] Hidalgo D.A (1989). Fibula free flap: a new method of mandible reconstruction. Plast Reconstr Surg.

[2] Newington D.P., Sykes P.J (1991). The versatility of the free fibula flap in the management of traumatic long bone defects. Injury.

[3] Taylor G.I., Miller G.D., Ham F.J (1975). The free vascularized bone graft. a clinical extension of microvascular techniques. Plast Reconstr Surg.

[4] Minami A., Kasashima T., Iwasaki N., Kato H., Kaneda K. (2000). Vascularised fibular grafts. an experience of 102 patients. J Bone Joint Surg Br.

[5] Zhang C., Sun J., Zhu H. (2015). Microsurgical free flap reconstructions of the head and neck region: shanghai experience of 34 years and 4640 flaps. Int J Oral Maxillofac Surg.

[6] Awad M.E., Altman A., Elrefai R., Shipman P., Looney S., Elsalanty M. (2019). The use of vascularized fibula flap in mandibular reconstruction; a comprehensive systematic review and meta-analysis of the observational studies. J Cranio-Maxillo-fac Surg Off Publ Eur Assoc Cranio-Maxillo-fac Surg.

[7] Shpitzer T., Neligan P., Boyd B., Gullane P., Gur E., Freeman J. (1997). Leg morbidity and function following fibular free flap harvest. Ann Plast Surg.

[8] Zimmermann C.E., Börner B.I., Hasse A., Sieg P. (2001). Donor site morbidity after microvascular fibula transfer. Clin Oral Investig.

[9] Bodde E.W.H., de Visser E., Duysens J.E.J., Hartman E.H.M (2003). Donor-site morbidity after free vascularized autogenous fibular transfer: subjective and quantitative analyses. Plast Reconstr Surg.

[10] Kanaya K., Wada T., Kura H., Yamashita T., Usui M., Ishii S. (2002). Valgus deformity of the ankle following harvesting of a vascularized fibular graft in children. J Reconstr Microsurg.

[11] Hidalgo D.A., Rekow A. (1995). A review of 60 consecutive fibula free flap mandible reconstructions. Plast Reconstr Surg.

[12] Tang C.L., Mahoney J.L., McKee M.D., Richards R.R., Waddell J.P., Louie B. (1998). Donor site morbidity following vascularized fibular grafting. Microsurgery.

[13] Chou S.W., Liao H.T., Yazar S., Lin C.H., Lin Y.C., Wei F.C (2009). Assessment of fibula osteoseptocutaneous flap donor-site morbidity using balance and gait test. J Orthop Res Off Publ Orthop Res Soc.

[14] Rendenbach C., Rashad A., Hansen L. (2018). Functional donor site morbidity longer than one year after fibula free flap: a prospective biomechanical analysis. Microsurgery.

[15] Shindo M., Fong B.P., Funk G.F., Karnell L.H (2000). The fibula osteocutaneous flap in head and neck reconstruction: a critical evaluation of donor site morbidity. Arch Otolaryngol Head Neck Surg.

[16] Xu Z.F., Bai S., Zhang Z.Q., Duan W.Y., Wang Z.Q., Sun C.F (2017). A critical assessment of the fibula flap donor site. Head Neck.

[17] Sieg P., Taner C., Hakim S.G., Jacobsen H.C (2010). Long-term evaluation of donor site morbidity after free fibula transfer. Br J Oral Maxillofac Surg.

[18] Lee J.H., Chung C.Y., Myoung H., Kim M.J., Yun P.Y (2008). Gait analysis of donor leg after free fibular flap transfer. Int J Oral Maxillofac Surg.

[19] Page M.J., McKenzie J.E., Bossuyt P.M. (2021). The PRISMA 2020 statement: an updated guideline for reporting systematic reviews. BMJ.

[20] Aromataris E., Lockwood C., Porritt K., Pilla B., Jordan Z. (2024). JBI manual for evidence synthesis. JBI.

[21] Prasad M. (2024). Introduction to the GRADE tool for rating certainty in evidence and recommendations. Clin Epidemiol Glob Health.

[22] Schardt C., Schmid A., Bodem J., Krisam J., Hoffmann J., Mertens C. (2017). Donor site morbidity and quality of life after microvascular head and neck reconstruction with free fibula and deep-circumflex iliac artery flaps. J Cranio-Maxillo-fac Surg Off Publ Eur Assoc Cranio-Maxillo-fac Surg.

[23] Rendenbach C., Kohlmeier C., Suling A. (2016). Prospective biomechanical analysis of donor-site morbidity after fibula free flap. J Cranio-Maxillo-fac Surg Off Publ Eur Assoc Cranio-Maxillo-fac Surg.

[24] Shah K.C., Peehal J.P., Shah A., Crank S., Flora H.S (2017). Star excursion balance test for assessment of dynamic instability of the ankle in patients after harvest of a fibular free flap: a two-centre study. Br J Oral Maxillofac Surg.

[25] Catalá-Lehnen P., Rendenbach C., Heiland M. (2012). Long-term donor-site morbidity after microsurgical fibular graft: is there a difference between the medial approach and the lateral approach?. J Oral Maxillofac Surg Off J Am Assoc Oral Maxillofac Surg.

[26] Ling X.F., Peng X., Samman N. (2013). Donor-site morbidity of free fibula and DCIA flaps. J Oral Maxillofac Surg Off J Am Assoc Oral Maxillofac Surg.

[27] Farhadi J., Valderrabano V., Kunz C., Kern R., Hinterman B., Pierer G. (2007). Free fibula donor-site morbidity: clinical and biomechanical analysis. Ann Plast Surg.

[28] Vittayakittipong P. (2013). Donor-site morbidity after fibula free flap transfer: a comparison of subjective evaluation using a visual analogue scale and point evaluation system. Int J Oral Maxillofac Surg.

[29] Ferrari S., Perlangeli G., Mammi P. (2018). Bilateral harvesting of a fibula free flap: assessment of morbidity. J Craniofac Surg.

[30] Maben D., Anehosur V., Kumar N. (2021). Assessment of donor site morbidity following fibula flap transfer. J Maxillofac Oral Surg.

[31] Attia S., Diefenbach J., Schmermund D. (2020). Donor-site morbidity after fibula transplantation in head and neck tumor patients: a split-leg retrospective study with focus on leg stability and quality of life. Cancers (Basel).

[32] Lin J.Y., Djohan R., Dobryansky M. (2009). Assessment of donor-site morbidity using balance and gait tests after bilateral fibula osteoseptocutaneous free flap transfer. Ann Plast Surg.

[33] Syczewska M., Krajewski R., Kirwil M., Szczerbik E., Kalinowska M. (2018). Gait changes in patients after reconstruction of facial bones with fibula and iliac crest free vascularized flaps. Acta Bioeng Biomech.

[34] Baj A., Lovecchio N., Bolzoni A., Mapelli A., Giannì A.B., Sforza C. (2015). Stair ascent and descent in assessing donor-site morbidity following osteocutaneous free fibula transfer: a preliminary study. J Oral Maxillofac Surg Off J Am Assoc Oral Maxillofac Surg.

[35] Pacifici I., Pallotta L., Bolzoni A., Beltramini G., Zago M., Sforza C. (2017). Gait performance in assessing donor-site morbidity following osteocutaneous free fibula transfer: a preliminary study. Gait Posture.

[36] Di Giuli R., Zago M., Beltramini G.A. (2019). Donor-site morbidity after osteocutaneous free fibula transfer: longitudinal analysis of gait performance. J Oral Maxillofac Surg Off J Am Assoc Oral Maxillofac Surg.

[37] Macdonald K.I., Mark Taylor S., Trites J.R.B. (2011). Effect of fibula free flap harvest on the gait of head and neck cancer patients: preliminary results. J Otolaryngol—Head Neck Surg J Oto-Rhino-Laryngol Chir Cervico-Faciale.

[38] Li P., Fang Q., Qi J., Luo R., Sun C. (2015). Risk factors for early and late donor-site morbidity after free fibula flap harvest. J Oral Maxillofac Surg Off J Am Assoc Oral Maxillofac Surg.

[39] Crosby M.A., Martin J.W., Robb G.L., Chang D.W (2008). Pediatric mandibular reconstruction using a vascularized fibula flap. Head Neck.

[40] Momoh A.O., Yu P., Skoracki R.J., Liu S., Feng L., Hanasono M.M (2011). A prospective cohort study of fibula free flap donor-site morbidity in 157 consecutive patients. Plast Reconstr Surg.

[41] Anthony J.P., Rawnsley J.D., Benhaim P., Ritter E.F., Sadowsky S.H., Singer M.I (1995). Donor leg morbidity and function after fibula free flap mandible reconstruction. Plast Reconstr Surg.

[42] Zavala A., Ore J.F., Broggi A., De Pawlikowski W. (2021). Pediatric mandibular reconstruction using the vascularized fibula free flap: functional outcomes in 34 consecutive patients. Ann Plast Surg.

[43] Sugiura Y., Sarukawa S., Hayasaka J., Kamochi H., Noguchi T., Mori Y. (2018). Mandibular reconstruction with free fibula flaps in the elderly: a retrospective evaluation. Int J Oral Maxillofac Surg.

[44] Santamaría E., Galaso-Trujillo J.R., Palafox D. (2021). Donor-site morbidity following free fibula flap harvest for mandibular or maxillary reconstruction in pediatric patients. J Craniofac Surg.

[45] Slijepcevic A.A., Wax M.K., Hanasono M. (2023). Post-operative outcomes in pediatric patients following facial reconstruction with fibula free flaps. Laryngoscope.

[46] Vail T.P., Urbaniak J.R (1996). Donor-site morbidity with use of vascularized autogenous fibular grafts. J Bone Joint Surg Am.

[47] Gupton M., Munjal A., Kang M. (2025).

[48] Pacelli L.L., Gillard J., McLoughlin S.W., Buehler M.J (2003). A biomechanical analysis of donor-site ankle instability following free fibular graft harvest. J Bone Joint Surg Am.

[49] Lang C.J., Frederick R.W., Hutton W.C (1998). A biomechanical study of the ankle syndesmosis after fibular graft harvest. J Spinal Disord.

[50] Yang L., Xu H.Z., Liang D.Z., Lu W., Zhong S.Z., Ouyang J. (2012). Biomechanical analysis of the impact of fibular osteotomies at tibiotalar joint: a cadaveric study. Indian J Orthop.

[51] Bozkurt M., Yavuzer G., Tönük E., Kentel B. (2005). Dynamic function of the fibula. gait analysis evaluation of three different parts of the shank after fibulectomy: proximal, middle and distal. Arch Orthop Trauma Surg.

[52] Feuvrier D., Sagawa Y., Béliard S., Pauchot J., Decavel P. (2016). Long-term donor-site morbidity after vascularized free fibula flap harvesting: clinical and gait analysis. J Plast Reconstr Aesthetic Surg.

[53] Warmerdam E., Horn D., Filip R., Freier K., Ganse B., Classen C. (2024). Gait asymmetries after fibular free flap harvest: a cross-sectional observational study. Clin Biomech Bristol Avon.

[54] Maurer-Ertl W., Glehr M., Friesenbichler J. (2012). No adverse affect after harvesting of free fibula osteoseptocutaneous flaps on gait function. Microsurgery.

[55] Ta N.H., Gao J., Philpott C. (2021). A systematic review to examine the relationship between objective and patient-reported outcome measures in sinonasal disorders: recommendations for use in research and clinical practice. Int Forum Allergy Rhinol.

[56] Wu Z., Xie P., Gu S. (2025). Clinical outcomes of arthroscopic modified suture augmentation versus internalbrace™ reconstruction in the treatment of chronic ankle instability. BMC Musculoskelet Disord.

[57] Kaikkonen A., Kannus P., Järvinen M. (1994). A performance test protocol and scoring scale for the evaluation of ankle injuries. Am J Sports Med.

[58] Park J., Han K. (2025). Quantifying gait asymmetry in stroke patients: a statistical parametric mapping (SPM) approach. Med Sci Monit Int Med J Exp Clin Res.

[59] Matsuda T., Fujino Y., Morisawa T. (2025). Reliability and validity examination of a new gait motion analysis system. Sensors.

[60] Barker S., Craik R., Freedman W., Herrmann N., Hillstrom H. (2006). Accuracy, reliability, and validity of a spatiotemporal gait analysis system. Med Eng Phys.

[61] Merriaux P., Dupuis Y., Boutteau R., Vasseur P., Savatier X. (2017). A study of VICON system positioning performance. Sensors.

[62] Ricciardi C., Pisani N., Donisi L. (2023). Agreement between optoelectronic system and wearable sensors for the evaluation of gait spatiotemporal parameters in progressive supranuclear palsy. Sensors.

