# Supplementary material for: Ankle instability and gait disturbance after free fibula flap reconstruction in head and neck cancer reconstruction: A systematic review
Source: JPRAS Open. 2025 Aug 7;46:33–49. doi: 10.1016/j.jpra.2025.08.005 (PMC12405634; doi:10.1016/j.jpra.2025.08.005)
Supplement: Supplementary file 1 [file mmc1.docx]

Supplementary table 1: The precise search strategy with the assistance of a health science librarian

All searches were run on 2024-02-06

**Ovid MEDLINE(R) ALL 1946 to February 05, 2024**
 **/** =Mesh-term

**exp** =exploded Mesh-term

**ab** =abstract

**kf =**keyword heading word

**ti** =title

| Search terms | | | Number of results |
| --- | --- | --- | --- |
| **Fibula flap** | | | |
|  | 1 | ((Fibula* adj4 harvest*) or (Fibula* adj4 free) or (Fibula* adj4 flap) or (Fibula* adj4 flaps) or (Fibula* adj4 transplant*) or (Fibula* adj4 transfer*) or (Fibula* adj4 graft*)).ab,kf,ti. | 4923 |
|  | 2 | exp Surgical Flaps/ | 69024 |
|  | 3 | (surgical flap* or flap surger*).ab,kf,ti. | 3745 |
|  | 4 | 2 OR 3 | 70631 |
|  | 5 | Fibula/ | 9818 |
|  | 6 | "fibula*".ab,kf,ti. | 15643 |
|  | 7 | 5 or 6 | 18873 |
|  | 8 | 4 and 7 | 2714 |
|  | 9 | 1 or 8 | 5418 |
| **Terms broadly related to balance and ancle stability** | | | |
|  | 10 | (gait* or walk* or balance* or Posture Equilibrium* or Postural Equilibrium* or body Equilibrium* or Musculoskeletal Equilibrium or kinesiotherapy or Postural Control* or Posture Control* or Mobilization or mobilisation or Remedial Exercise* or Exercise Therap* or Rehabilitation Exercise* or Occupational Therap* or Ergotherap* or "activities of daily living" or rehab* or Habilitation or "recovery of function" or physiotherap* or "physical therap*" or stability or instability or ambulation or movement* or Motion or Joint Flexibility or motor).ab,kf,ti. | 2377686 |
|  | 11 | exp Gait/ or exp Walking/ or exp Postural Balance/ or exp Early Ambulation/ or exp Exercise Therapy/ or exp Occupational Therapy/ or Rehabilitation/ or exp Physical Therapy Modalities/ or exp Movement/ or exp "Range of Motion, Articular"/ or exp Motor Activity/ | 898542 |
|  | 12 | 10 or 11 | 2923702 |
| **Sets combined** | | | |
|  | 13 | 9 and 12 | 1389 |
| **Limit to English language** | | | |
|  | 14 | limit 13 to english language | 1254 |

**Embase** (Embase.com)

**/de** =Emtree term

**/exp** =exploded Emtree term

**:ti,ab,kw** =title, abstract, keyword

| Search terms | | | Number of results |
| --- | --- | --- | --- |
| **Fibula flap** | | | |
|  | 1 | ((fibula* NEAR/4 harvest*):ti,ab,kw) OR ((fibula* NEAR/4 free):ti,ab,kw) OR ((fibula* NEAR/4 flap):ti,ab,kw) OR ((fibula* NEAR/4 flaps):ti,ab,kw) OR ((fibula* NEAR/4 transplant*):ti,ab,kw) OR ((fibula* NEAR/4 transfer*):ti,ab,kw) OR ((fibula* NEAR/4 graft*):ti,ab,kw) | 5616 |
|  | 2 | 'fibula graft'/exp | 2592 |
|  | 3 | 'surgical flaps'/exp | 25076 |
|  | 4 | 'surgical flap*':ti,ab,kw OR 'flap surger*':ti,ab,kw | 4908 |
|  | 5 | #3 OR #4 | 28475 |
|  | 6 | 'fibula'/exp | 11660 |
|  | 7 | fibula*:ti,ab,kw | 19185 |
|  | 8 | #6 OR #7 | 23102 |
|  | 9 | #5 AND #8 | 2198 |
|  | 10 | #1 OR #2 OR #9 | 6534 |
| **Terms broadly related to balance and ancle stability** | | | |
|  | 11 | gait*:ti,ab,kw OR walk*:ti,ab,kw OR balance*:ti,ab,kw OR 'posture equilibrium*':ti,ab,kw OR 'postural equilibrium*':ti,ab,kw OR 'body equilibrium*':ti,ab,kw OR 'musculoskeletal equilibrium':ti,ab,kw OR kinesiotherapy:ti,ab,kw OR 'postural control*':ti,ab,kw OR 'posture control*':ti,ab,kw OR mobilization:ti,ab,kw OR mobilisation:ti,ab,kw OR 'remedial exercise*':ti,ab,kw OR 'exercise therap*':ti,ab,kw OR 'rehabilitation exercise*':ti,ab,kw OR 'occupational therap*':ti,ab,kw OR ergotherap*:ti,ab,kw OR 'activities of daily living':ti,ab,kw OR rehab*:ti,ab,kw OR habilitation:ti,ab,kw OR 'recovery of function':ti,ab,kw OR physiotherap*:ti,ab,kw OR 'physical therap*':ti,ab,kw OR stability:ti,ab,kw OR instability:ti,ab,kw OR ambulation:ti,ab,kw OR movement*:ti,ab,kw OR motion:ti,ab,kw OR 'joint flexibility':ti,ab,kw OR motor:ti,ab,kw | 3004615 |
|  | 12 | 'walking'/exp | 143485 |
|  | 13 | 'body equilibrium'/exp | 24625 |
|  | 14 | 'mobilization'/exp | 40317 |
|  | 15 | 'kinesiotherapy'/exp | 106287 |
|  | 16 | 'occupational therapy'/exp | 29620 |
|  | 17 | 'rehabilitation'/exp | 514133 |
|  | 18 | 'physiotherapy'/exp | 117418 |
|  | 19 | 'movement (physiology)'/exp | 541999 |
|  | 20 | 'motor dysfunction'/exp | 989501 |
|  | 21 | #11 OR #12 OR #13 OR #14 OR #15 OR #16 OR #17 OR #18 OR #19 OR #20 | 4232217 |
| **Sets combined** | | | |
|  | 22 | #10 AND #21 | 2055 |
| **Exclusion of conference abstracts and limit to English language** | | | |
|  | 23 | #22 NOT 'conference abstract'/it | 1784 |
|  | 24 | #22 NOT 'conference abstract'/it AND [english]/lim | 1614 |

**Cinahl**

**MH** = CINAHL heading. The plus (+) indicates that the heading is exploded.

**TI** = title

**AB** = abstract

| Search terms | | | Number of results |
| --- | --- | --- | --- |
| **Fibula flap** | | | |
|  | S1 | TI ( (Fibula* N3 harvest*) or (Fibula* N3 free) or (Fibula* N3 flap) or (Fibula* N3 flaps) or (Fibula* N3 transplant*) or (Fibula* N3 transfer*) or (Fibula* N3 graft*) ) OR AB ( (Fibula* N3 harvest*) or (Fibula* N3 free) or (Fibula* N3 flap) or (Fibula* N3 flaps) or (Fibula* N3 transplant*) or (Fibula* N3 transfer*) or (Fibula* N3 graft*) ) | 1,026 |
|  | S2 | (MH "Surgical Flaps+") | 8,069 |
|  | S3 | TI ( "Surgical flap*" OR "flap surger*" ) OR AB ( "Surgical flap*" OR "flap surger*" ) | 487 |
|  | S4 | S2 OR S3 | 8,279 |
|  | S5 | (MH "Fibula") | 1,906 |
|  | S6 | TI Fibula* OR AB Fibula* | 4,206 |
|  | S7 | S5 OR S6 | 4,643 |
|  | S8 | S4 AND S7 | 415 |
|  | S9 | S1 OR S8 | 1,113 |
| **Terms broadly related to balance and ancle stability** | | | |
|  | S10 | TI ( gait* or walk* or balance* or "Posture Equilibrium*" or "Postural Equilibrium*" or "body Equilibrium*" or "Musculoskeletal Equilibrium" or kinesiotherapy or "Postural Control*" or "Posture Control*" or Mobilization or mobilisation or "Remedial Exercise*" or "Exercise Therap*" or "Rehabilitation Exercise*" or "Occupational Therap*" or Ergotherap* or "activities of daily living" or rehab* or Habilitation or "recovery of function" or physiotherap* or "physical therap*" or stability or instability or ambulation or movement* or Motion or "Joint Flexibility" or motor ) OR AB ( gait* or walk* or balance* or "Posture Equilibrium*" or "Postural Equilibrium*" or "body Equilibrium*" or "Musculoskeletal Equilibrium" or kinesiotherapy or "Postural Control*" or "Posture Control*" or Mobilization or mobilisation or "Remedial Exercise*" or "Exercise Therap*" or "Rehabilitation Exercise*" or "Occupational Therap*" or Ergotherap* or "activities of daily living" or rehab* or Habilitation or "recovery of function" or physiotherap* or "physical therap*" or stability or instability or ambulation or movement* or Motion or "Joint Flexibility" or motor ) | 528,841 |
|  | S11 | (MH "Gait+") OR (MH "Walking+") OR (MH "Balance, Postural+") OR (MH "Early Ambulation") OR (MH "Therapeutic Exercise+") OR (MH "Occupational Therapy+") OR (MH "Rehabilitation") OR (MH "Physical Therapy+") OR (MH "Movement+") OR (MH "Range of Motion") OR (MH "Motor Activity+") OR (MH "Joint Mobilization") OR (MH "Kinesiotherapy") | 316,572 |
|  | S12 | S10 OR S11 | 693,130 |
| **Sets combined** | | | |
|  | S13 | S9 AND S12 | 329 |
| **Limit to English language** | | | |
|  | S14 | S9 AND S12 Limiters- English language | 326 |

**PEDro**, **the Physiotherapy Evidence Database,** avalible at https://pedro.org.au/

*The database was last updated on 5 February 2024 (this includes records added or amended since 4 December 2023)*

| Search terms | | | Number of results |
| --- | --- | --- | --- |
| **The following search was run in the title & abstract field** | | | |
|  | 1 | Fibula* | 37 |

Note: you are supposed to be able to run complex search strategies in PEDro, but after running a few tests and concluded that the syntax did not work quite as the help section stated. Therefore, we resorted to a simple truncated single word search that returned anything in the database mentioning **fibula** in the title or abstract. This “better safe than sorry” approach was possible since the database is fairly small and does not return a lot of results anyway.
