# Supplementary material for: Ankle instability and gait disturbance after free fibula flap reconstruction in head and neck cancer reconstruction: A systematic review
Source: JPRAS Open. 2025 Aug 7;46:33–49. doi: 10.1016/j.jpra.2025.08.005 (PMC12405634; doi:10.1016/j.jpra.2025.08.005)
Supplement: Supplementary file 2 [file mmc2.docx]

*Supplementary Table 2: Critical Appraisal Results for Included Studies Using JBI Critical Appraisal Checklist for Case series*

| **Study** | **Q1** | **Q2** | **Q3** | **Q4** | **Q5** | **Q6** | **Q7** | **Q8** | **Q9** | **Q10** | | **Overall risk** | **Overall risk (%)** |
| --- | --- | --- | --- | --- | --- | --- | --- | --- | --- | --- | --- | --- | --- |
| 1. Pacifici et al. 2017 | Y | Y | N | U | Y | N | N | Y | U Y | |  | - | 50 |
| 2. Shpitzer et al. 1997 | Y | U | Y | Y | N | Y | Y | Y | Y U | |  | - | 70 |
| 3. Di Giuli et al. 2018 | Y | Y | Y | Y | Y | Y | Y | Y | Y Y | |  | + | 100 |
| 4. Li et al. 2015 | Y | Y | Y | Y | Y | Y | Y | Y | Y Y | |  | + | 100 |
| 5. Sieg et al. 2010 | N | U | U | U | U | N | Y | Y | Y Y | |  | X | 40 |
| 6. Rendenbach et al. 2016 | Y | Y | Y | Y | U | Y | Y | Y | Y Y | |  | + | 90 |
| 7. Farhadi et al. 2007 | Y | Y | Y | Y | N | Y | Y | Y | Y Y | |  | + | 90 |
| 8. Ling et al. 2013 | Y | U | Y | Y | N | Y | Y | Y | Y Y | |  | + | 80 |
| 9. Ferrari et al. 2018 | Y | Y | Y | Y | Y | Y | Y | Y | U NA | |  | + | 80 |
| 10. Crosby et al. 2008 | Y | Y | Y | Y | N | Y | Y | Y | Y N | |  | + | 80 |

*Note.* JBI = Joanna Briggs Institute; Y = yes; N = no; U = unclear; NA = not applicable;

The total quality score between 0-100%, were 71-100% = (low risk); 50-70% = (moderate risk) and 0-50% = (high risk). + = low risk, - moderate risk and x = high risk.

*Questions of JBI Checklist for Case Series*

*Q1. Were there clear criteria for inclusion in the case series?*

*Q2. Was the condition measured in a standard, reliable way for all participants included in the case series?*

*Q3. Were valid methods used for identification of the condition for all participants included in the case series?*

*Q4. Did the case series have consecutive inclusion of participants?*

*Q5. Did the case series have complete inclusion of participants?*

*Q6. Was there clear reporting of the demographics of the participants in the study?*

*Q7. Was there clear reporting of clinical information of the participants?*

*Q8. Were the outcomes or follow-up results of cases clearly reported?*

*Q9. Was there clear reporting of the presenting site(s)/clinic(s) demographic information?*

*Q10. Was statistical analysis appropriate?*

Munn Z, Barker T, Moola S, Tufanaru C, Stern C, McArthur A, Stephenson M, Aromataris E. Methodological quality of case series studies, JBI Evidence Synthesis, doi: 10.11124/JBISRIR-D-19-00099
