# Supplementary material for: Ankle instability and gait disturbance after free fibula flap reconstruction in head and neck cancer reconstruction: A systematic review
Source: JPRAS Open. 2025 Aug 7;46:33–49. doi: 10.1016/j.jpra.2025.08.005 (PMC12405634; doi:10.1016/j.jpra.2025.08.005)
Supplement: Supplementary file 3 [file mmc3.docx]

*Supplementary table 3: Critical Appraisal Results for Included Studies Using JBI Critical Appraisal Checklist for Case Control Studies.*

| **Study** | **Q1** | **Q2** | **Q3** | **Q4** | **Q5** | **Q6** | **Q7** | **Q8** | **Q9** | **Q10** | **Overall risk** | **Overall risk (%)** |
| --- | --- | --- | --- | --- | --- | --- | --- | --- | --- | --- | --- | --- |
| 1. Lin et al. 2009 | Y | Y | Y | Y | Y | N | NA | Y | Y | Y | + | 80 |

*Note.* JBI = Joanna Briggs Institute; Y = yes; N = no; U = unclear; NA = not applicable;

The total quality score between 0-100%, were 71-100% = (low risk); 50-70% = (moderate risk) and 0-50% = (high risk). + = low risk, - moderate risk and x = high risk.

*Questions of JBI Checklist for Case Control Studies*

*Q1. Were the groups comparable other than presence of disease in cases or absence of disease in controls?*

*Q2. Were cases and controls matched appropriately?*

*Q3. Were the same criteria used for identification of cases and controls?*

*Q4. Was exposure measured in a standard, valid and reliable way?*

*Q5. Was exposure measured in the same way for cases and controls?*

*Q6. Were confounding factors identified?*

*Q7. Were strategies to deal with confounding factors stated?*

*Q8. Were outcomes assessed in a standard, valid and reliable way for cases and controls?*

*Q9. Was the exposure period of interest long enough to be meaningful?*

*Q10. Was appropriate statistical analysis used?*

Moola S, Munn Z, Tufanaru C, Aromataris E, Sears K, Sfetcu R, Currie M, Qureshi R, Mattis P, Lisy K, Mu P-F. Chapter 7: Systematic reviews of etiology and risk . In: Aromataris E, Munn Z (Editors). JBI Manual for Evidence Synthesis. JBI, 2020. Available from https://synthesismanual.jbi.global
