# Supplementary material for: Ankle instability and gait disturbance after free fibula flap reconstruction in head and neck cancer reconstruction: A systematic review
Source: JPRAS Open. 2025 Aug 7;46:33–49. doi: 10.1016/j.jpra.2025.08.005 (PMC12405634; doi:10.1016/j.jpra.2025.08.005)
Supplement: Supplementary file 4 [file mmc4.docx]

| **Cohort Study** | **Q1** | **Q2** | **Q3** | **Q4** | **Q5** | **Q6** | **Q7** | **Q8** | **Q9** | **Q10** | **Q11** | **Overall risk** | **Overall risk (%)** |
| --- | --- | --- | --- | --- | --- | --- | --- | --- | --- | --- | --- | --- | --- |
| 1. Xu et al. 2017 | Y | Y | Y | N | N | Y | Y | Y | U | N | U | - | 54,6 |
| 2. Lee et al. 2008 | NA | NA | Y | N | N | Y | Y | Y | Y | N | Y | - | 54,6 |
| 3. Anthony et al. 1995 | Y | Y | Y | N | N | Y | N | Y | Y | N | Y | - | 63,7 |
| 4. Shindo et al. 2000 | Y | Y | U | Y | U | NA | Y | Y | U | U | Y | - | 54,6 |
| 5. Rendenbach et al. 2018 | Y | Y | Y | Y | Y | NA | Y | Y | U | U | Y | + | 73 |
| 6. Zavala et al. 2021 | Y | Y | Y | U | U | NA | U | Y | Y | NA | Y | - | 54,6 |
| 7. Zimmerman et al. 2001 | Y | NA | NA | Y | Y | Y | Y | Y | Y | Y | Y | + | 81,9 |
| 8. Momoh et al. 2011 | Y | NA | NA | Y | U | Y | Y | Y | Y | Y | Y | + | 73 |
| 9. Syczewska et al 2018 | Y | Y | Y | N | U | Y | Y | Y | U | U | Y | + | 73 |
| 10. Macdonald et al. 2011 | Y | Y | Y | N | N | Y | Y | Y | Y | Y | Y | + | 81,9 |
| 11. Sugiura et al. 2018 | Y | Y | Y | N | U | Y | Y | U | Y | N | Y | - | 63,7 |
| 12. Di Guili et al. 2019 | U | NA | Y | U | U | Y | Y | Y | NA | NA | Y | - | 68 |

*Supplementary table 4: Critical Appraisal Results for Included Studies Using JBI Critical Appraisal Checklist for Cohort Studies*

*Note.* JBI = Joanna Briggs Institute; Y = yes; N = no; U = unclear; NA = not applicable;

The total quality score between 0-100%, were 71-100% = (low risk); 50-70% = (moderate risk) and 0-50% = (high risk). + = low risk, - moderate risk and x = high risk.

*Questions of JBI Checklist for Cohort Studies*

Q1: Were the two groups similar and recruited from the same population?

Q2: Were the exposures measured similarly to assign people to both exposed and unexposed groups?

Q3: Was the exposure measured in a valid and reliable way?

Q4: Were confounding factors identified?

Q5: Were strategies to deal with confounding factors stated?

Q6: Were the groups/participants free of the outcome at the start of the study (or at the moment of exposure)?

Q7: Were the outcomes measured in a valid and reliable way?

Q8: Was the follow up time reported and sufficient to be long enough for outcomes to occur?

Q9: Was follow up complete, and if not, were the reasons to loss to follow up described and explored?

Q10: Were strategies to address incomplete follow up utilized?

Q11: Was appropriate statistical analysis used?

Moola S, Munn Z, Tufanaru C, Aromataris E, Sears K, Sfetcu R, Currie M, Qureshi R, Mattis P, Lisy K, Mu P-F. Chapter 7: Systematic reviews of etiology and risk. In: Aromataris E, Munn Z (Editors). *Joanna Briggs Institute Reviewer's Manual*. The Joanna Briggs Institute, 2017. Available from https://reviewersmanual.joannabriggs.org/
