# Supplementary material for: Ankle instability and gait disturbance after free fibula flap reconstruction in head and neck cancer reconstruction: A systematic review
Source: JPRAS Open. 2025 Aug 7;46:33–49. doi: 10.1016/j.jpra.2025.08.005 (PMC12405634; doi:10.1016/j.jpra.2025.08.005)
Supplement: Supplementary file 5 [file mmc5.docx]

*Supplementary table 5: Critical Appraisal Results for Included Studies Using JBI Critical Appraisal Checklist for Analytical Cross-Sectional studies.*

| **Study** | **Q1** | **Q2** | **Q3** | **Q4** | **Q5** | **Q6** | **Q7** | **Q8** | **Overall risk** | **Overall risk (%)** |
| --- | --- | --- | --- | --- | --- | --- | --- | --- | --- | --- |
| 1. Baj et al. 2015 | Y | Y | Y | Y | NA | NA | Y | Y | + | 75 |
| 2. Schardt et al. 2017 | Y | Y | Y | Y | NA | NA | Y | Y | + | 75 |
| 3.Vittayakittipong et al. 2013 | Y | Y | Y | Y | N | N | U | Y | - | 62,5 |
| 4. Santamaría et al. 2021 | Y | Y | Y | Y | Y | Y | Y | Y | + | 100 |
| 5. Slijepcevic et al. 2023 | Y | Y | Y | Y | NA | NA | Y | Y | + | 75 |
| 6. Shah et al. 2017 | Y | Y | Y | Y | NA | NA | Y | Y | + | 75 |
| 7. Catalá-Lehnen et al. 2012 | Y | Y | Y | Y | NA | NA | Y | Y | + | 75 |
| 8. Attia et al. 2020 | Y | Y | Y | Y | NA | NA | Y | Y | + | 75 |
| 9. Maben et al. 2021 | Y | Y | Y | Y | NA | NA | Y | U | - | 62,5 |

*Note.* JBI = Joanna Briggs Institute; Y = yes; N = no; U = unclear; NA = not applicable;

The total quality score between 0-100%, were 71-100% = (low risk); 50-70% = (moderate risk) and 0-50% = (high risk). + = low risk, - moderate risk and x = high risk.

*Questions of JBI Checklist for Analytical cross-sectional studies*

Q1: Were the criteria for inclusion in the sample clearly defined?

Q2: Were the study subjects and the setting described in detail?

Q3: Was the exposure measured in a valid and reliable way?

Q4: Were objective, standard criteria used for measurement of the condition?

Q5: Were confounding factors identified?

Q6: Were strategies to deal with confounding factors stated?

Q7: Were the outcomes measured in a valid and reliable way?

Q8: Was appropriate statistical analysis used?

Moola S, Munn Z, Tufanaru C, Aromataris E, Sears K, Sfetcu R, Currie M, Qureshi R, Mattis P, Lisy K, Mu P-F. Chapter 7: Systematic reviews of etiology and risk . In: Aromataris E, Munn Z (Editors). JBI Manual for Evidence Synthesis. JBI, 2020.
