# Supplementary material for: Ankle instability and gait disturbance after free fibula flap reconstruction in head and neck cancer reconstruction: A systematic review
Source: JPRAS Open. 2025 Aug 7;46:33–49. doi: 10.1016/j.jpra.2025.08.005 (PMC12405634; doi:10.1016/j.jpra.2025.08.005)
Supplement: Supplementary file 6 [file mmc6.docx]

| Study | **Risk of bias** | **Inconsistency** | **Indirectness** | **Imprecision** | **Publication bias** | **Quality of evidence** |
| --- | --- | --- | --- | --- | --- | --- |
| 1. (Shindo m.fl. 2000) | Moderate | Low | Low | Moderate | Moderate | Moderate |
| 2. (Lin m.fl. 2009) | Low | Low | Moderate | NA (Low) | Low | Moderate |
| **3. (Rendenbach m.fl. 2018) Inlogg** | Low | High | Moderate | Low | Moderate | Moderate |
| **4. (Zavala m.fl. 2021)** | Moderate | High | Moderate | Low | Moderate | Moderate |
| **5. (Zimmermann m.fl. 2001)** | Low | Low | Moderate | Moderate | High | Moderate |
| **6. (Momoh m.fl. 2011)** | Low | Moderate | Moderate | High | Moderate | Moderate |
| 7. (Baj m.fl. 2015) | Low | Moderate | Low | Low | Moderate | Low |
| **8. (Schardt m.fl. 2017)** | Low | Moderate | Moderate | Moderate | Moderate | Moderate |
| 9. (Anthony m.fl. 1995) | Moderate | Moderate | Moderate | Moderate | Moderate | Moderate |
| **10. (Lee m.fl. 2008)** | Moderate | Low | Moderate | Low | Moderate | Moderate |
| **11. (Xu m.fl. 2017)** | Moderate | Moderate | Moderate | Low | Moderate | Moderate |
| 12. (Syczewska m.fl. 2018) | Low | Moderate | Moderate | Moderate | Moderate | Moderate |
| **13. (Macdonald m.fl. 2011)** | Moderate | Low | Low | High | NA | Low |
| **14. (Vittayakittipong 2013)** | Moderate | Moderate | Low | Low | High | Moderate |
| **15. (Pacifici m.fl., u.å.)** | Moderate | Low | Low | Low | Moderate | Low |
| **16. (Shpitzer m.fl. 1997)** | Moderate | Low | Low | High | NA | Low |
| **17. (Rendenbach m.fl. 2016)** | Low | Moderate | Moderate | Low | Moderate | Moderate |
| **18. (Sieg m.fl. 2010)** | High | Moderate | Moderate | Moderate | High | Moderate |
| **19. (Li m.fl. 2015)** | Low | High | Moderate | Moderate | High | Moderate |
| 20. (Di Giuli m.fl. 2019) | Low | High | High | Low | High | Moderate |
| **21. (Sugiura m.fl. 2018)** | Low | Moderate | Moderate | Moderate | High | Moderate |
| **22. (Santamaría m.fl. 2021)** | Low | Moderate | Moderate | Low | Low | Low |
| **23. (Slijepcevic m.fl. 2023)** | Low | Low | Moderate | Moderate | Low | Low |
| **24. (Shah m.fl. 2017)** | Low | Moderate | Moderate | Moderate to low | High | Moderate |
| **25. (Catalá-Lehnen m.fl. 2012)** | Low | Moderate | Moderate | Low | High | Moderate |
| **26.(Ferrari m.fl. 2018)** | Low | Moderate | Low | Low to Very low | Moderate | Low |
| **27. (Crosby m.fl. 2008)** | Low | Moderate | Low | Low | High | Moderate |
| 28. (Attia m.fl. 2020) | Low | Low to moderate | Moderate | Moderate | High | Moderate |
| 29. (Maben, Anehosur, och Kumar 2021) | Moderate | High | Moderate | Low | High | Moderate |
| **30. (Ling, Peng, och Samman 2013)** | Low | Moderate | Moderate | Low | High | Moderate |
| **31. (Farhadi m.fl. 2007)** | Low | High | Moderate | Low | High | Moderate |
| **32. (Chou m.fl. 2009)** | Moderate | High | Moderate | Low | High | Moderate |

Supplementary table 6: The certainty of the evidence assessed using the Grading of Recommendations Assessment, Development, and Evaluation (GRADE) tool, classified into four levels: high, moderate, low, or very low.
